# Supplementary material for: Clonal dynamics of alloreactive T cells in kidney allograft rejection after anti-PD-1 therapy
Source: Nat Commun. 2023 Mar 21;14:1549. doi: 10.1038/s41467-023-37230-4 (PMC10027853; doi:10.1038/s41467-023-37230-4)
Supplement: Supplementary file 1 — Supplementary Information [file 41467_2023_37230_MOESM1_ESM.pdf]

A B C

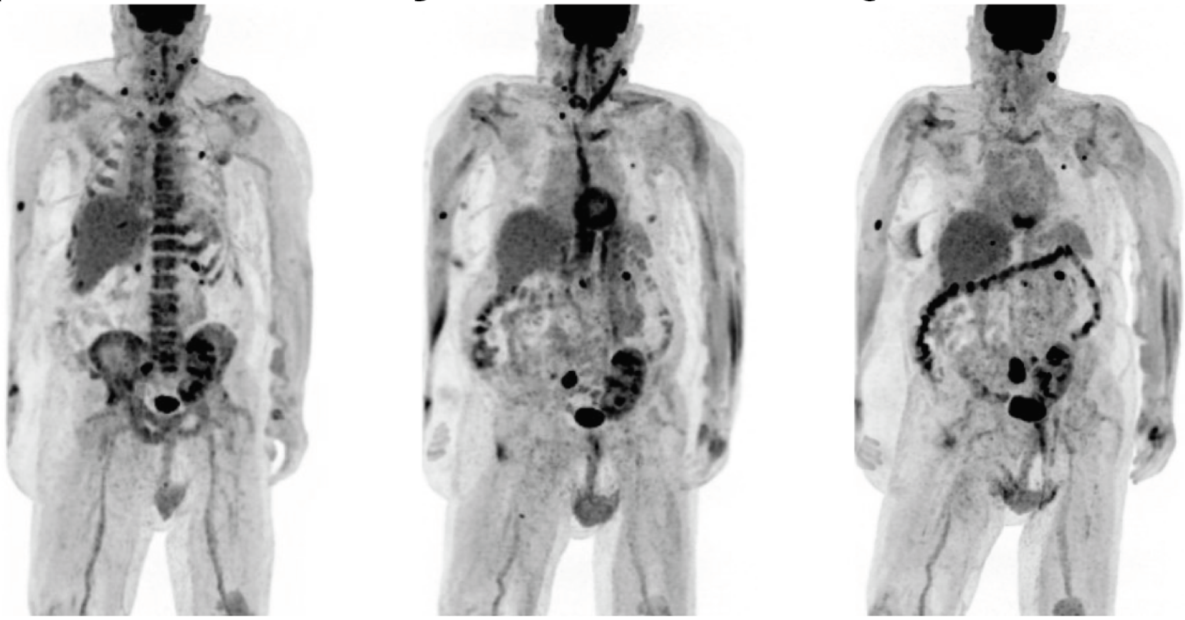

Supplementary Fig. 1. Representative PET scan images obtained 2 weeks prior to treatment with pembrolizumab **(A)**, 3 months after pembrolizumab initiation **(B)**, and 6 months after pembrolizumab initiation **(C)** are shown. Between (A) and (B), there was interval mixed response with new FDG-avid cervical lymph nodes and persistent or increasing FDG uptake in some bone lesions and periportal lymph nodes and decrease in extent and intensity of other bone lesions. There was stable intense cutaneous uptake in the left neck. Between (B) and (C), there was a mixed response with the appearance of a new FDG-avid left axillary node and decreased FDG uptake in the multiple lymph nodes. There was persistent cutaneous uptake in the left neck.

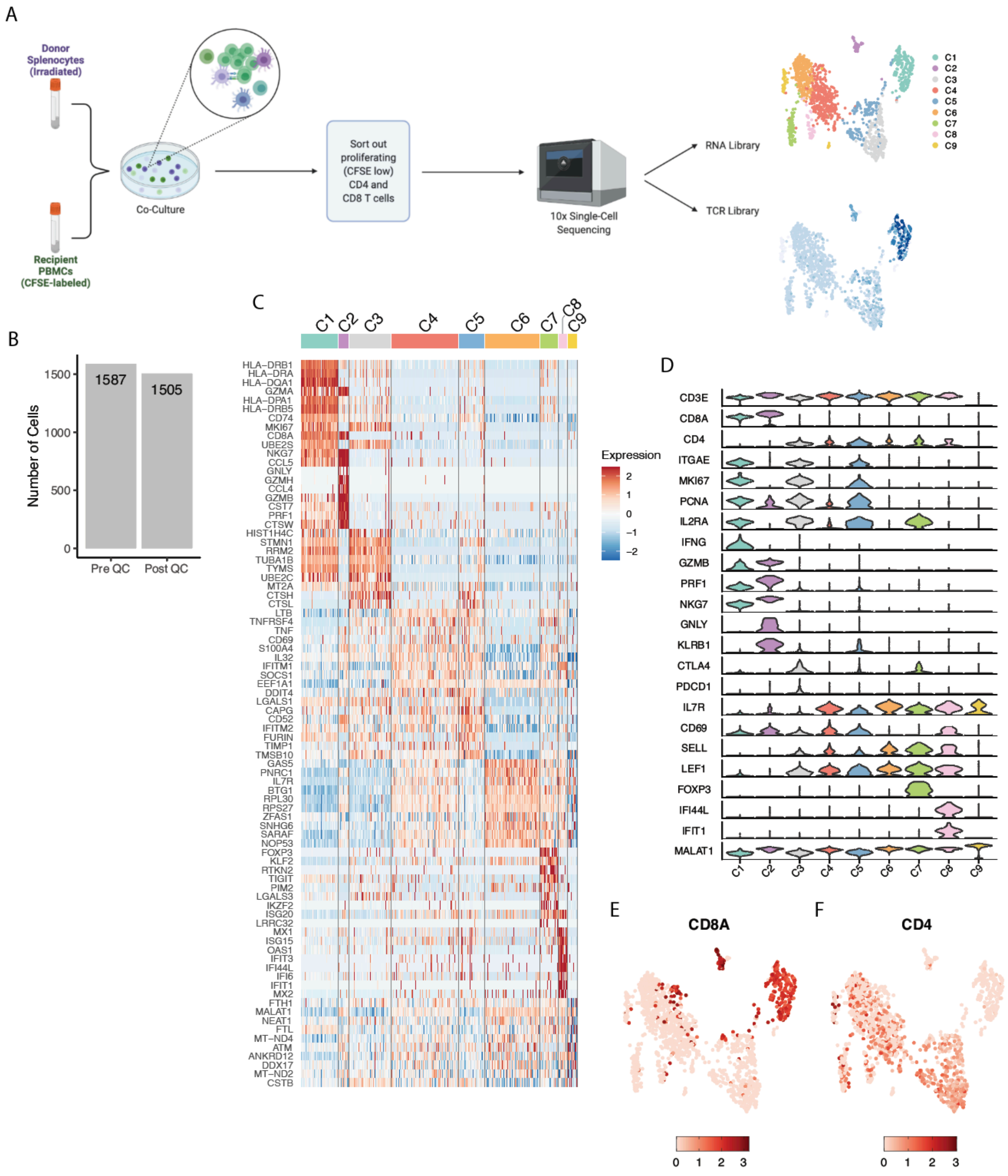

Supplementary Fig. 2. A. Experimental schematic of mixed lymphocyte reaction. **B.** Bar plot of the number of single-cell transcriptomes before and after quality control filtering. **C.** Heatmap of the differentially expressed genes per cluster. **D.** Violin plot of cluster markers identified in single-cell RNA sequencing (scRNAseq). UMAPs of the expression of *CD8A* **(E)** and *CD4* **(F)**. Schematic in **A** created with BioRender.com.

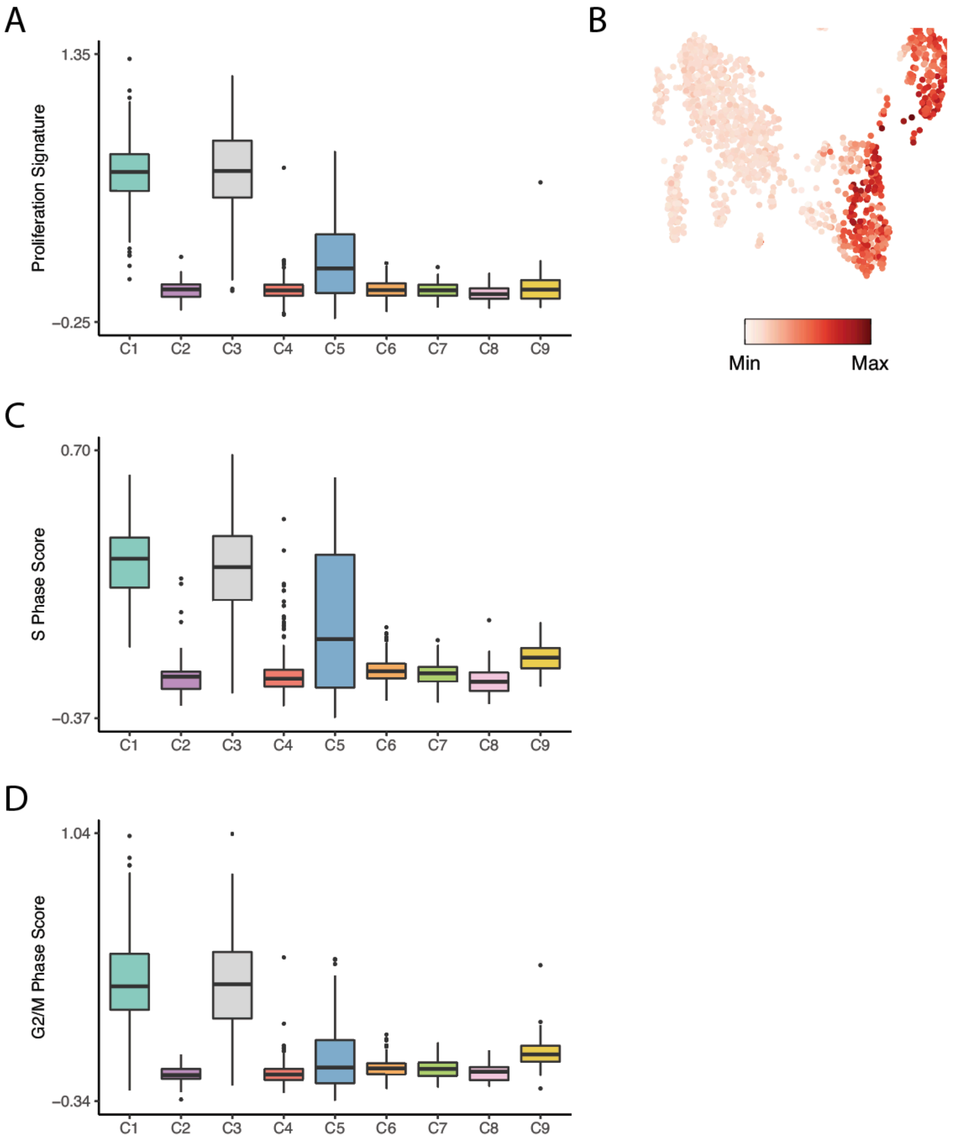

Supplementary Fig. 3. A. Box plot of proliferation signature from Li, et al 2019. **B.** UMAP of proliferation signature. Box plots of the gene expression score of genes associated with the S phase **(C)** or the G2/M phase **(D)** of the cell cycle, used to generate predicted cell cycle stages in **Figure 2B**. Boxes in **A, C,** and **D** denote the interquartile range, with the horizontal line at the median, and outlier cells are shown as dots.

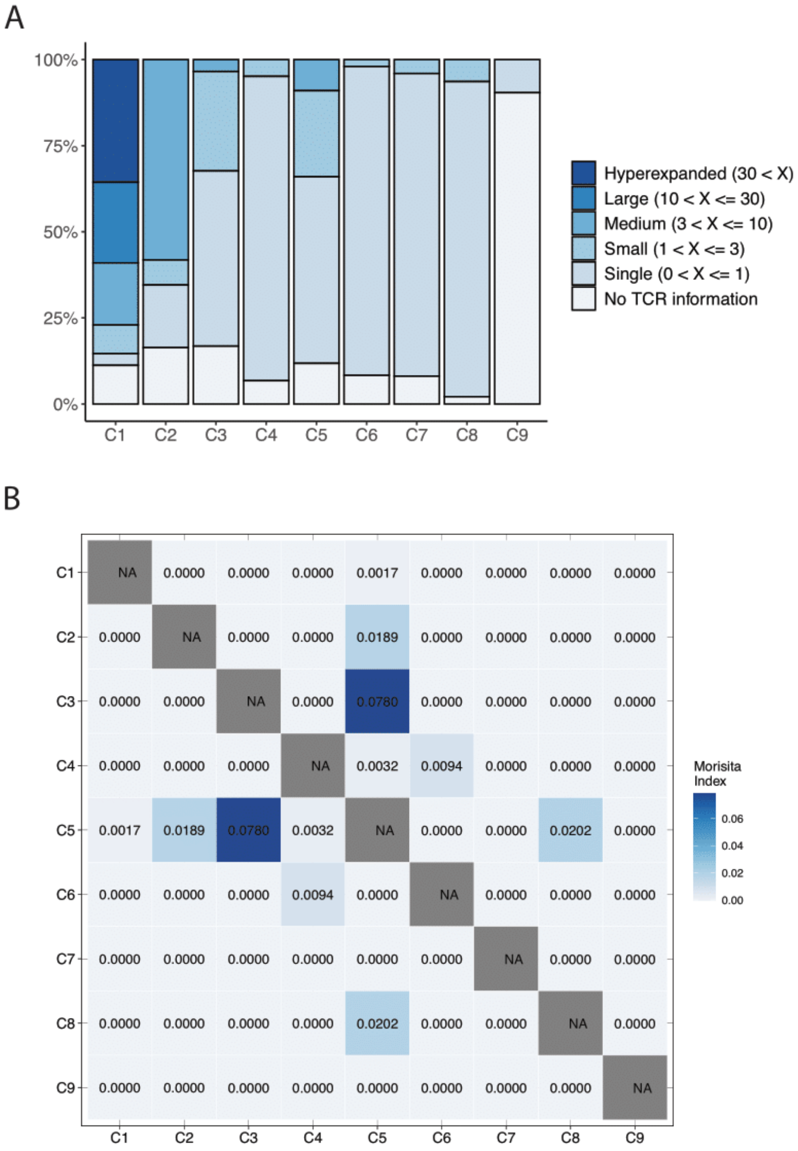

Supplementary Fig. 4. A. Bar plot of clonal expansion across clusters, by category. **B.** Heat map of clonal sharing by Morisita overlap index between scRNAseq clusters.

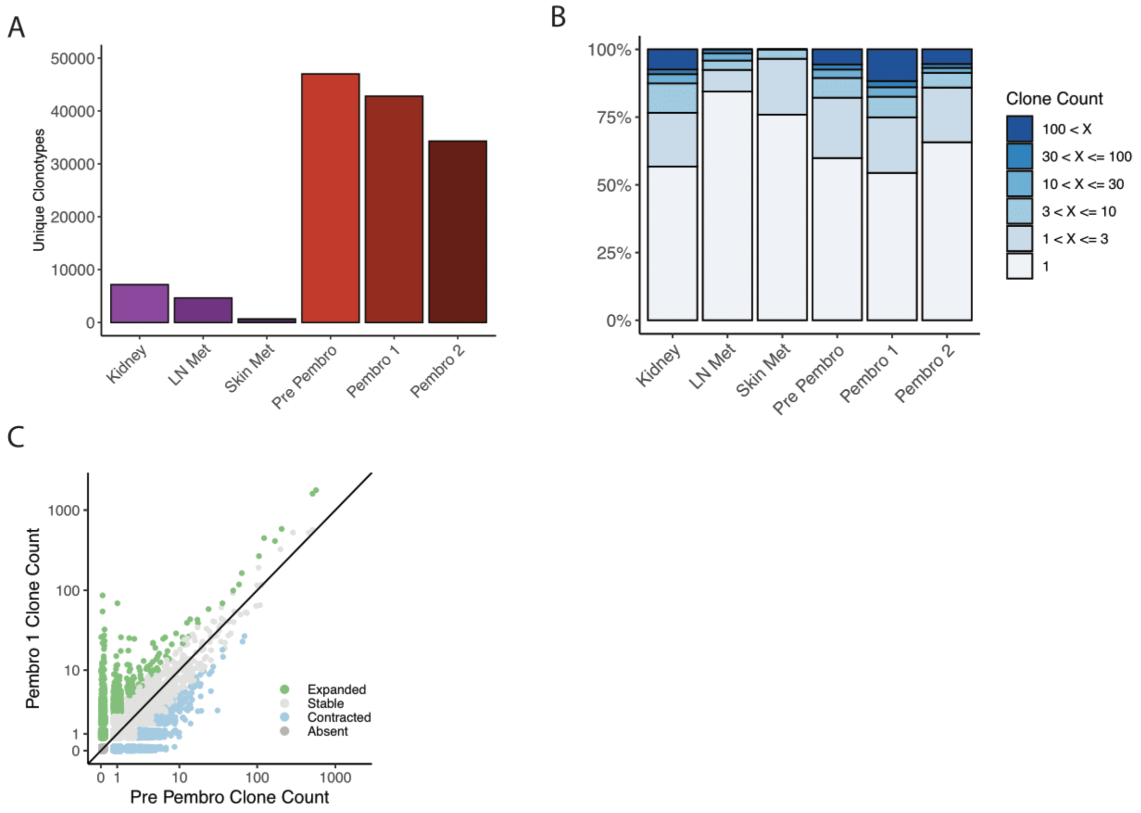

Supplementary Fig. 5. A. Bar plot of the number of unique clonotypes obtained from each sample. **B.** Bar plot of clonal expansion across samples. **C.** Scatter plot of the number of cells from each clone before and after pembrolizumab. Clones (dots) are colored by expansion or contraction of clone size.

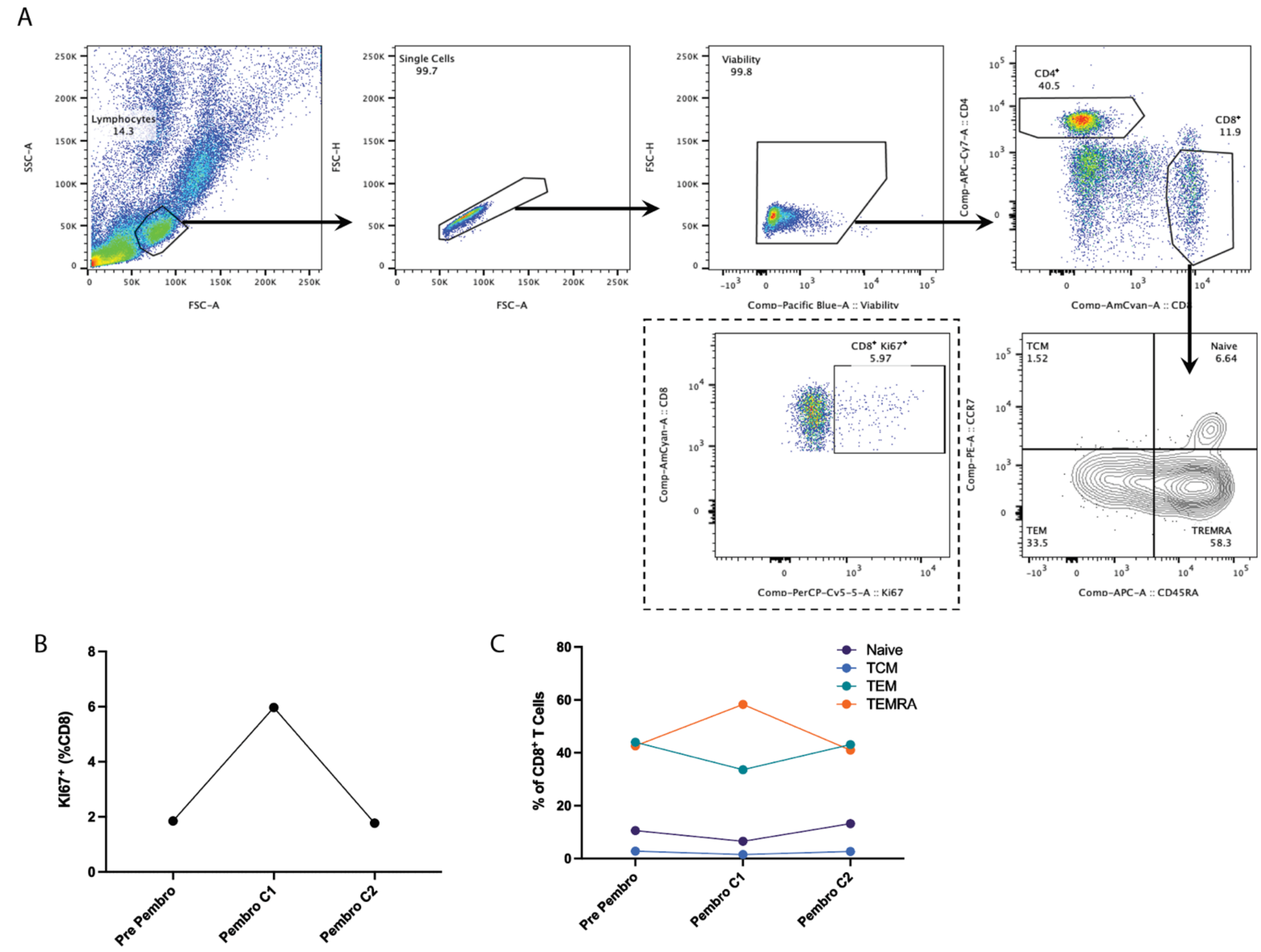

Supplementary Fig. 6. A. Schematic of flow-cytometry sorting used in **B** and **C**. **B.** Quantification of the percent of $CD8^+$ T cells found to be $Ki67^+$ in PBMC samples over treatment period. **C.** Quantification of the distribution of $CD8^+$ T cell subsets in PBMC samples over treatment period. TCM, T central memory; TEM, T effector memory; TEMRA, T effector memory recently activated.

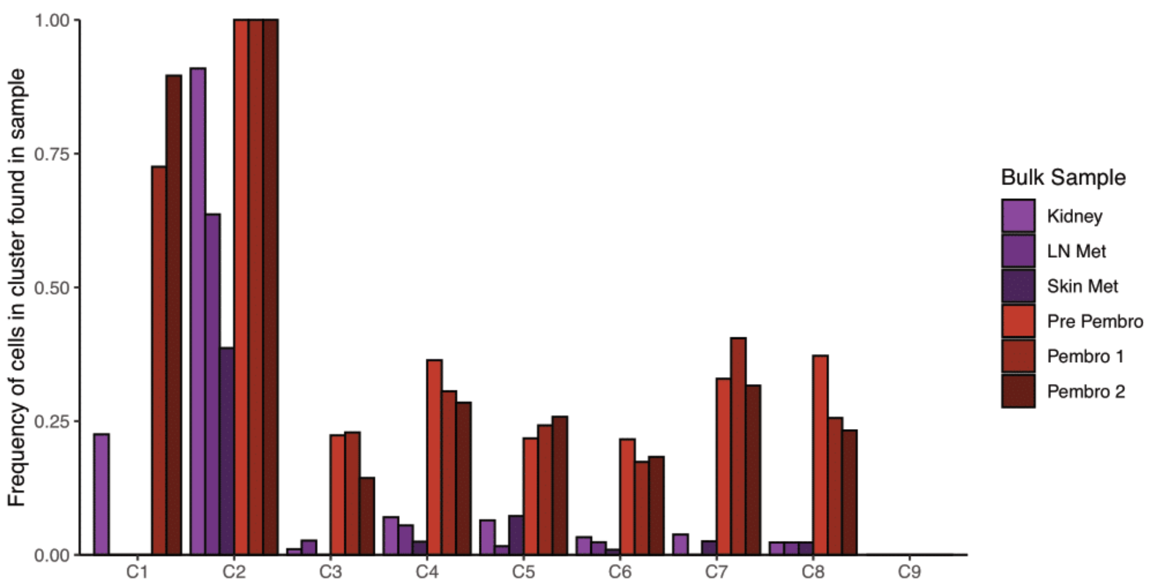

Supplementary Fig. 7. Bar plot of the frequency of bulk TCR clones found to be matched across scRNAseq clusters.

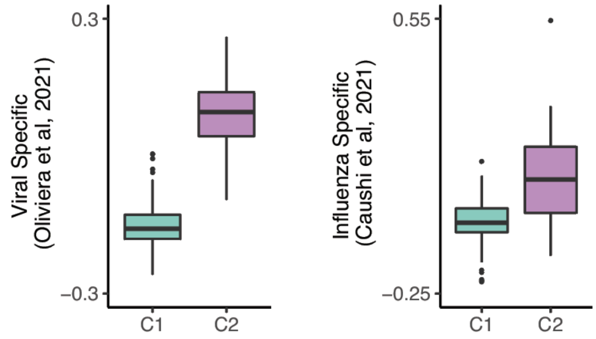

Supplementary Fig. 8. Box plot of viral- and Influenza-specific gene signatures for C1 and C2 clones. Boxes denote the interquartile range, with the horizontal line at the median, and outlier cells are shown as dots.

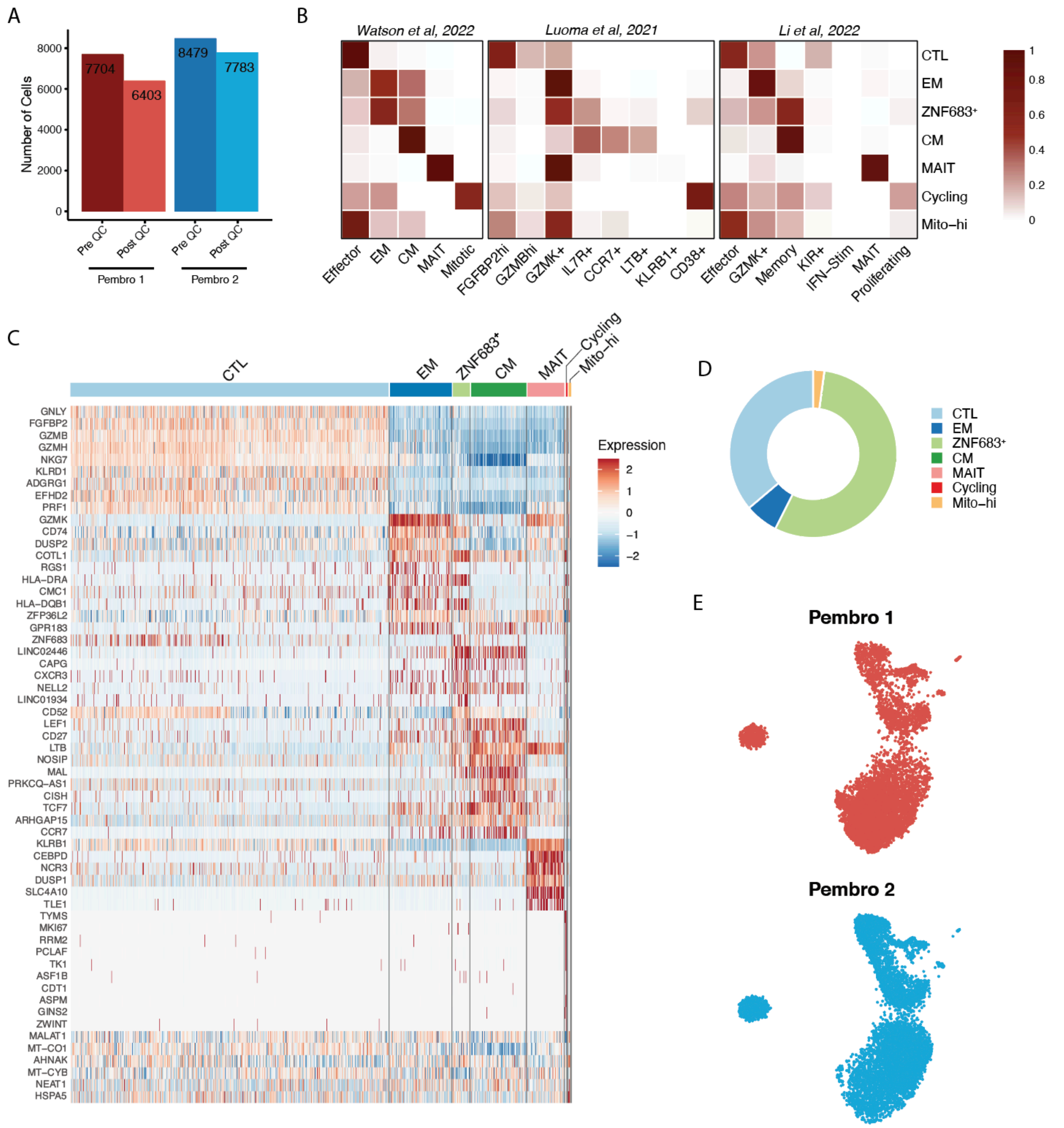

Supplementary Fig. 9. A. Bar plot of the number of single-cell transcriptomes before and after quality control filtering, per timepoint. **B.** Heatmap of the reference mapping results using other blood CD8^{+} T cell datasets. **C.** Heatmap of the differentially expressed genes per cluster. **D.** Donut plot of the re-mapping of the Cycling cluster. **E.** UMAPs split by timepoint.
