# Supplementary material for: Clonal dynamics of alloreactive T cells in kidney allograft rejection after anti-PD-1 therapy
Source: Nat Commun. 2023 Mar 21;14:1549. doi: 10.1038/s41467-023-37230-4 (PMC10027853; doi:10.1038/s41467-023-37230-4)
Supplement: Supplementary file 3 — Description of Additional Supplementary Files [file 41467_2023_37230_MOESM3_ESM.pdf]

Description of Additional Supplementary Files

Supplementary Data 1. Differentially expressed genes for each MLR cluster. Statistical calculations made using a two-sided Wilcoxon test with Bonferroni correction.

Supplementary Data 2. Gene lists of proliferation, S phase, G2/M phase, viral-specific, Influenza-specific, and activation signatures.

Supplementary Data 3. Comparison of largest cluster C2 clone to McPAS-TCR database results found within a Levenshtein distance of 1. Bolded items represent a match between this TCR and previously identified TCRs.

Supplementary Data 4. Differentially expressed genes for each blood non-naive CD8 cluster. Statistical calculations made using a two-sided Wilcoxon test with Bonferroni correction.

Supplementary Data 5. Markers used in flow-cytometry panel.
