# Supplementary material for: Clonal dynamics of alloreactive T cells in kidney allograft rejection after anti-PD-1 therapy
Source: Nat Commun. 2023 Mar 21;14:1549. doi: 10.1038/s41467-023-37230-4 (PMC10027853; doi:10.1038/s41467-023-37230-4)
Supplement: Supplementary file 9 — Reporting Summary [file 41467_2023_37230_MOESM9_ESM.pdf]

nature portfolio

Corresponding author(s): Deepak Rao and Naoka Murakami

Last updated by author(s): 01/31/2023

Reporting Summary

Nature Portfolio wishes to improve the reproducibility of the work that we publish. This form provides structure for consistency and transparency in reporting. For further information on Nature Portfolio policies, see our Editorial Policies and the Editorial Policy Checklist.

Statistics

For all statistical analyses, confirm that the following items are present in the figure legend, table legend, main text, or Methods section.

n/a	Confirmed	
x		The exact sample size (*n*) for each experimental group/condition, given as a discrete number and unit of measurement
x		A statement on whether measurements were taken from distinct samples or whether the same sample was measured repeatedly
	x	The statistical test(s) used AND whether they are one- or two-sided<br>*Only common tests should be described solely by name; describe more complex techniques in the Methods section.*
x		A description of all covariates tested
	x	A description of any assumptions or corrections, such as tests of normality and adjustment for multiple comparisons
	x	A full description of the statistical parameters including central tendency (e.g. means) or other basic estimates (e.g. regression coefficient) AND variation (e.g. standard deviation) or associated estimates of uncertainty (e.g. confidence intervals)
	x	For null hypothesis testing, the test statistic (e.g. *F*, *t*, *r*) with confidence intervals, effect sizes, degrees of freedom and *P* value noted<br>*Give P values as exact values whenever suitable.*
x		For Bayesian analysis, information on the choice of priors and Markov chain Monte Carlo settings
x		For hierarchical and complex designs, identification of the appropriate level for tests and full reporting of outcomes
x		Estimates of effect sizes (e.g. Cohen's *d*, Pearson's *r*), indicating how they were calculated

Our web collection on statistics for biologists contains articles on many of the points above.

Software and code

Policy information about availability of computer code

Data collection	The samples were analyzed by flow cytometry (FACS Canto-II, BD) immediately after staining using FACSDiva software (BD, v9.0). The data were analyzed by FlowJo software (BD, v10), and data was graphed using GraphPad Prism (v9).
Data analysis	Single-cell sequencing data processing was performed using CellRanger (v6.0.1), and downstream single-cell and TCR sequencing analysis was completed in R (v4.0.3) using Seurat (v4.0.2) and scRepertoire (v.1.1.4).<br><br>Scripts used for the analyses and figure generation of this paper are available at https://github.com/dunlapg/transplant-rejection-ICI.

For manuscripts utilizing custom algorithms or software that are central to the research but not yet described in published literature, software must be made available to editors and reviewers. We strongly encourage code deposition in a community repository (e.g. GitHub). See the Nature Portfolio guidelines for submitting code & software for further information.

Data

Policy information about availability of data

All manuscripts must include a data availability statement. This statement should provide the following information, where applicable:

- Accession codes, unique identifiers, or web links for publicly available datasets
- A description of any restrictions on data availability
- For clinical datasets or third party data, please ensure that the statement adheres to our policy

The processed single-cell RNA/TCR and bulk TCR sequencing data used in this study are available in the NCBI Gene Expression Omnibus (GEO) database under accession code GSE216763 [https://www.ncbi.nlm.nih.gov/geo/query/acc.cgi?acc=GSE216763]. The bulk TCR sequencing data used in this study are also available in the Adaptive Biotechnologies ImmuneACCESS portal [https://doi.org/10.21417/2022NC].

Human research participants

Policy information about studies involving human research participants and Sex and Gender in Research.

Reporting on sex and gender	The manuscript describes the outcomes of a single (male) patient.
Population characteristics	The manuscript describes the outcomes of a single patient, age 77.
Recruitment	This patient was included due to receiving a kidney transplant before undergoing checkpoint inhibitor therapy.
Ethics oversight	The biobank study has been approved by Dana Farber Cancer Institute Institutional Review Board (IRB 05-042). Donor splenocytes were cryopreserved in 10% DMSO-FCS and archived at BWH Tissue Typing Laboratory (IRB 2021P003483).

Note that full information on the approval of the study protocol must also be provided in the manuscript.

Field-specific reporting

Please select the one below that is the best fit for your research. If you are not sure, read the appropriate sections before making your selection.

[x] Life sciences [ ] Behavioural & social sciences [ ] Ecological, evolutionary & environmental sciences

For a reference copy of the document with all sections, see nature.com/documents/nr-reporting-summary-flat.pdf

Life sciences study design

All studies must disclose on these points even when the disclosure is negative.

Sample size	This paper describes the analysis and outcomes of a single patient, with samples collected at multiple points in time and from multiple tissue locations.
Data exclusions	No data were excluded from this analysis.
Replication	The patient's outcomes are not subject to replication as it is a report of a single case.
Randomization	Because this analysis included a single patient, randomization could not be applied.
Blinding	Because this analysis included a single patient, blinding could not be applied.

Reporting for specific materials, systems and methods

We require information from authors about some types of materials, experimental systems and methods used in many studies. Here, indicate whether each material, system or method listed is relevant to your study. If you are not sure if a list item applies to your research, read the appropriate section before selecting a response.

Materials & experimental systems

n/a	Involved in the study
[ ]	[x] Antibodies
[x]	[ ] Eukaryotic cell lines
[x]	[ ] Palaeontology and archaeology
[x]	[ ] Animals and other organisms
[x]	[ ] Clinical data
[x]	[ ] Dual use research of concern

Methods

n/a	Involved in the study
[x]	[ ] ChIP-seq
[ ]	[x] Flow cytometry
[x]	[ ] MRI-based neuroimaging

Antibodies

Antibodies used

Marker Clone Company Catalog# Dilution
CD3 OKT3 Biolegend 317336 1:200
CD4 OKT4 Biolegend 317450 1:200
CD8a SK1 Biolegend 980904 1:200
CD45RA HI100 Biolegend 304150 1:100
CD25 M-A251 Biolegend 356134 1:50
CD127 A019D5 Biolegend 351322 1:50
CXCR5 J252D4 Biolegend 356914 1:50
CCR7 G043H7 Biolegend 353204 1:40
CXCR3 G025H7 Biolegend 353704 1:50
CCR6 C034E3 Biolegend 353418 1:40
CD11c 3.9 Biolegend 301614 1:50
CD14 M5E2 Biolegend 301842 1:50
CD16 3G8 Biolegend 980104 1:100
HLA-DR 5.1H11 Biolegend 307618 1:100
CD19 HIB19 Biolegend 302254 1:100
IgG HP6017 Biolegend 409310 1:50
IgD IA6-2 Biolegend 348234 1:100
IgM MHM-88 Biolegend 314514 1:100
CD38 HB-7 Biolegend 356608 1:100
CD27 O323 Biolegend 302810 1:20
IFN-g 4S.B3 Biolegend 502530 1:40
IL-4 8D4-8 eBioscience 17-7049-42 1:40
IL-17 eBio64DEC17 eBioscience 12-7179-42 1:40

Validation

All antibodies used in this study were obtained from reputable commercial vendors.

Biolegend: "Antibody clones are then tested in a variety of assays to see which applications they are suited for....Thus, the clone cross-validates itself by demonstrating functionality across orthogonal testing methods."

eBioscience: "A diverse portfolio of high-quality primary and secondary antibodies is available ... as well as validated for multiple applications, including flow cytometry, western blotting, and immunoprecipitation."

Flow Cytometry

Plots

Confirm that:

- [x] The axis labels state the marker and fluorochrome used (e.g. CD4-FITC).
- [x] The axis scales are clearly visible. Include numbers along axes only for bottom left plot of group (a 'group' is an analysis of identical markers).
- [x] All plots are contour plots with outliers or pseudocolor plots.
- [x] A numerical value for number of cells or percentage (with statistics) is provided.

Methodology

Sample preparation

Flow cytometry:
PBMCs were thawed and stained with fixable viability dye (Thermo Fisher) for 30 minutes on ice, followed by surface staining in 2% FCS-PBS with various antibodies for 30 minutes on ice. For intracellular cytokine staining, the cells were stimulated with phorbol myristate acetate (PMA) and ionomycin in the presence of GolgiStop (BD) for 4 hours at 37C, 5% CO2, and stained with cell surface markers, followed by permeabilization with Foxp3 staining kit (eBioscience) and intracellular staining.

Flow sorting (MLR):

	Donor splenocytes were gamma-irradiated (30 Gy) and were loaded with CellTrace Violet dye (Invitrogen) to be able to distinguish donor and recipient cells. 1 x10^6 recipient PBMC (Pembro C2 timepoint) were loaded with CFSE (Invitrogen) and co-cultured with 4 x10^6 irradiated donor splenocytes for 5 days in 10% FCS-RPMI, supplemented with recombinant human IL-2 (20 units/ml, PeproTech) and anti-CD28 (1 µg/ml) at 37C and 5% CO2.
Instrument	Flow cytometry:<br>FACS Canto-II (BD)<br><br>Flow sorting (MLR):<br>FACS Aria (BD)
Software	FlowJo (BD), Prism (GraphPad)
Cell population abundance	Flow sorting (MLR):<br>The strategy below resulted in 0.5% (>3400 events) of the total population being sorted for single-cell sequencing.
Gating strategy	Flow cytometry:<br>FSC/SSC was used for gating lymphocytes from the PBMC population. FSC-A/FSC-H was used to gate single cells, and viable cells were further gated using Pacific Blue. CD4/CD8 were then used to gate CD4+ and CD8+ populations, and further CCR7/CD45RA were used to gate naive, Tem, Tcm, and Temra populations.<br><br>Flow sorting (MLR):<br>Sorting was accomplished on viable, CellTrace violet-, CD3+, and CFSE low gating.

☒ Tick this box to confirm that a figure exemplifying the gating strategy is provided in the Supplementary Information.
